# Application of Community Detection Algorithm to Investigate the Correlation between Imaging Biomarkers of Tumor Metabolism, Hypoxia, Cellularity, and Perfusion for Precision Radiotherapy in Head and Neck Squamous Cell Carcinomas

**DOI:** 10.3390/cancers13153908

**Published:** 2021-08-03

**Authors:** Ramesh Paudyal, Milan Grkovski, Jung Hun Oh, Heiko Schöder, David Aramburu Nunez, Vaios Hatzoglou, Joseph O. Deasy, John L. Humm, Nancy Y. Lee, Amita Shukla-Dave

**Affiliations:** 1 Department of Medical Physics, Memorial Sloan Kettering Cancer Center, New York, NY 10065, USA; paudyalr@mskcc.org (R.P.); grkovskm@mskcc.org (M.G.); OhJ@mskcc.org (J.H.O.); aramburd@mskcc.org (D.A.N.); DeasyJ@mskcc.org (J.O.D.); hummj@mskcc.org (J.L.H.); 2 Department of Radiology, Memorial Sloan Kettering Cancer Center, New York, NY 10065, USA; schoderh@mskcc.org (H.S.); HatzoglV@mskcc.org (V.H.); 3 Department of Radiation Oncology, Memorial Sloan Kettering Cancer Center, New York, NY 10065, USA; leen2@mskcc.org

**Keywords:** positron emission tomography, diffusion-weighted, dynamic contrast-enhanced, kinetic modeling, fast exchange regime model, community detection algorithm, spin-glass model

## Abstract

**Simple Summary:**

Integration of multimodality imaging (MMI) methods in head and neck squamous cell carcinomas (HNSCC) provides complementary information of the tumor and its microenvironment. Quantitative positron emission tomography (PET)/computed tomography (CT), DW- and DCE-MRI provide the functional information of tumor tissue based on metabolic process, diffusion of water molecules, and enhancement of water proton relaxation with a contrast agent, respectively. The present study aimed to investigate correlations at pre-treatment between quantitative imaging metrics derived from FDG-PET/CT(SUL), FMISO-PET/CT (K_1_, k_3_, TBR, and DV), DW-MRI (ADC, IVIM [D, D*, and f]), and FXR DCE-MRI [K^trans^, v_e_, and τ_i_]) using a community detection algorithm (CDA) based on the “spin-glass model” and Spearman rank analysis in patients with HNSCC. Correlations between MMI-derived quantitative metrics evaluated using a CDA in addition to the Spearman analysis in a larger population may enable the identification of potential biomarkers for prognostication and management of patients with HNSCC.

**Abstract:**

The present study aimed to investigate the correlation at pre-treatment (TX) between quantitative metrics derived from multimodality imaging (MMI), including ^18^F-FDG-PET/CT, ^18^F-FMISO-PET/CT, DW- and DCE-MRI, using a community detection algorithm (CDA) in head and neck squamous cell carcinoma (HNSCC) patients. Twenty-three HNSCC patients with 27 metastatic lymph nodes underwent a total of 69 MMI exams at pre-TX. Correlations among quantitative metrics derived from FDG-PET/CT (SUL), FMSIO-PET/CT (K_1_, k_3_, TBR, and DV), DW-MRI (ADC, IVIM [D, D*, and f]), and FXR DCE-MRI [K^trans^, v_e_, and τ_i_]) were investigated using the CDA based on a “spin-glass model” coupled with the Spearman’s rank, ρ, analysis. Mean MRI T_2_ weighted tumor volumes and SUL_mean_ values were moderately positively correlated (ρ = 0.48, *p* = 0.01). ADC and D exhibited a moderate negative correlation with SUL_mean_ (ρ ≤ −0.42, *p* < 0.03 for both). K_1_ and K^trans^ were positively correlated (ρ = 0.48, *p* = 0.01). In contrast, K^trans^ and k_3max_ were negatively correlated (ρ = −0.41, *p* = 0.03). CDA revealed four communities for 16 metrics interconnected with 33 edges in the network. DV, K^trans^, and K_1_ had 8, 7, and 6 edges in the network, respectively. After validation in a larger population, the CDA approach may aid in identifying useful biomarkers for developing individual patient care in HNSCC.

## 1. Introduction

Head and neck squamous cell carcinoma (HNSCC) is a complex disease with remarkable intratumoral heterogeneity resulting in different treatment responses and outcomes [1]. HNSCC arises from the mucosa lining of the aerodigestive tract, including the oropharyngeal axis. Human papillomavirus (HPV)-related oropharyngeal cancers (OPCs) have molecular features and etiology distinct from those of smoking- and alcohol-related HNSCC [2,3]. Both qualitative and quantitative imaging, including computed tomography (CT), T_1_-weighted and T_2_-weighted magnetic resonance imaging (MRI), positron emission tomography (PET)/CT, diffusion-weighted (DW-), and dynamic contrast-enhanced (DCE)-MRI, have shown potential in staging, predicting treatment (TX) response, and post-TX follow-up of patients with HNSCC [4,5,6,7,8].

Quantitative analysis of multimodality imaging (MMI), including ^18^F-Fluorodeoxyglucose (FDG) PET/CT, ^18^F-Fluoromisonidazole (FMISO) PET/CT, DW- and DCE-MRI, data provide imaging metrics, reflecting the tumor metabolism, hypoxia, cellularity, and vessel permeability in HNSCC [6,9,10,11,12]. Therefore, the measurement of MMI-derived quantitative imaging (QI) metrics at pre-TX is vital for evaluating and planning precision radiotherapy in HNSCC. The standardized uptake value (SUV) from ^18^F-FDG-PET/CT assesses the changes in glucose uptake as a measure of response to radiotherapy (RT) [13]. Pharmacokinetic modeling of FMISO yields a metric, a biomarker of cell oxygenation (hypoxia), reflecting malignant tissue radiosensitivity [14]. Previous studies have reported that pre-TX ^18^F-FMISO-PET/CT could aid in predicting RT outcome and survival prognosis in HNSCC [9,15]. Riaz et al. recently demonstrated that dose de-escalation of radiotherapy to 30 Gy based on intra-treatment hypoxia using imaging response utilizing ^18^F-FMISO-PET/CT was feasible, safe, and associated with minimal toxicity [16].

The measurement of diffusion of water molecules in malignant tissue can reveal abnormalities of the tissue cellular organization and microstructure [17]. The ADC derived from monoexponential modeling of diffusion-weighted (DW) signal data with at least two b-values, a surrogate marker of tumor cellularity, has shown promise in predicting and detecting early response to chemo-RT HNSCC in metastatic lymph nodes (LNs) [18,19]. Quantitative imaging (QI) metrics derived from the intravoxel incoherent motion (IVIM) model [20] without contrast agent (CA), including perfusion fraction (f) and true diffusion coefficient (D), exhibited potential markers for early prediction of chemo-RT response in HNSCC patients [21,22,23]. Paudyal et al. further reported subtypes within human papillomavirus-positive (HVP+) patients with HNSCC treated with 70 Gy chemo-RT. This finding raises the question of whether every individual should be treated with the same dose of radiation [23].

DCE-MRI pharmacokinetics modeling estimates perfusion/permeability and volume fractions of the CA distribution spaces based on the changes in the time course of signal intensity from target tissue after a bolus administration of CA [24]. The post-TX DCE-MRI showed potential for identifying residual masses, both in primary tumors and in metastatic LNs, that had failed [25]. The extended Tofts model [24], assuming an infinitesimally fast water exchange kinetics between the tissue compartments, derived volume transfer constant (K^trans^), extravascular extracellular volume fraction [EES] (v_e_), and plasma volume fraction (v_p_) from primary tumors and metastatic LNs have shown promise in differentiating responders from non-responders [26]. Shukla-Dave et al. reported that the skewness of pre-TX K^trans^ values was the strongest predictor of progression-free survival and overall survival in Stage IV HNSCC patients with the nodal disease [27]. Kim et al. implemented the fast exchange (FXR) model, accounting for the finite rate of transcytolemmal water exchange, and reported that the pre-TX K^trans^ exhibited a potential to predict metastatic LNs treatment response to chemo-RT in HNSCC cancer patients [28]. The poor pre-TX tumor perfusion may be a common mechanism associated with radioresistance and the development of the distant metastatic phenotype [29]. Recent preclinical and clinical studies suggested that the FXR model-derived intracellular water molecule’s mean lifetime (τ_i_) can be a surrogate marker of tumor cell metabolic activity [30,31]. Chawla et al. reported that the metric τ_i_ could be a prognostic marker in HNSCC patients [32].

Previous studies explored the correlation between metastatic LN tumor volume and ^18^F-FDG- PET/CT and ^18^F-FMISO-PET/CT derived QI metrics [15,33,34]. The correlations between ADC and ^18^F-FDG SUV results were inconsistent in HNSCC [33,34,35]. The mean K^trans^ and SUV_max_ showed a trend towards a significant positive correlation in 28 primary tumors of HNSCC [11]. Jansen reported significantly lower median K^trans^ and the rate constant of CA from the EES back into the plasma space, k_ep_, values in hypoxic than in non-hypoxic nodes in HNSCC [36]. Wiedenmann demonstrated that the multiple parameters’ values differ significantly between hypoxic and non-hypoxic tumor regions, defined on FMISO-PET/CT in HNSCC [37].

A Spearman correlation analysis between MMI-derived QI metrics measures the strength of a monotonic relationship. Still, it does not explicitly show how and to what extent these metrics are interconnected within a group. These QI metrics can be represented as a network in which nodes (metrics) with similar characteristics are clustered to form sub-networks (communities) [38]. Herein, the community detection algorithm (CDA) based on a “spin-glass model” was employed to create a community for MMI-derived metrics [39]. In the network, nodes within the same groups are densely coupled. In contrast, nodes between the group’s nodes are sparsely connected, indicating the CDA approach can be helpful to identify the cancer biomarkers for understanding solid tumor biology. To our knowledge, this is the first study that introduces a CDA-based “spin-glass model” approach in patients with HNSCC.

Despite the significant advances in MMI methods, identifying useful QI metrics that can assess the effectiveness of RT response in patients with HNSCC is still a challenging task. We hypothesize that the CDA approach could help identify robust biomarkers in developing cutting-edge strategies for precision therapy in HNSCC patients. The present study aimed to investigate correlations between QI metrics derived from MMI methods using a CDA based on the “spin-glass model” in HNSCC patients.

## 2. Materials and Methods

### 2.1. Patient Selection

Our institutional review board approved this prospective study compliant with the Health Insurance Portability and Accountability Act. We obtained written informed consent from all eligible patients who had a biopsy-proven, newly diagnosed HN cancer; diagnostic biopsies were tested for human papillomavirus (HPV) status before the CT and MRI study. Patients with previous chemotherapy or radiation therapy planned for upfront surgery and other primaries than HNSCC were excluded from the study. Between December 2013 and November 2015, a total of twenty-three (N = 23) HPV (21 HPV positive [+]  and 2 HPV negative [−]) HN cancer patients (median age = 58 years, range = 45–82 years; Male/Female = 21/2) enrolled in the study and underwent a total of 69 pre-TX examinations, including ^18^F-FDG-PET/CT (N = 23), ^18^F-FMISO dynamic PET/CT (N = 23), and MRI (combined DW- and DCE-MRI; N = 23). Of the 23 patients included with HNSCC, 15 patients had tumor sites in the base of the tongue, seven patients with tumors in a tonsil, one patient had an unknown primary tumor site, and four patients had bilateral metastatic LNs. The patients were categorized according to the American Joint Committee on Cancer (AJCC) tumor, node, metastasis (TNM) system. The majority of patients had T2 (65%), N2 nodal mass was found in all 23 patients, and none had M0. Patients were treated with concurrent chemotherapy and radiotherapy (70 Gy).

### 2.2. PET Data Acquisition

Baseline FDG-PET scans on HNSCC patients were performed for radiotherapy planning purposes.

Patients were positioned on a flat-top couch wearing a customized radiotherapy treatment immobilization mask, which allows for accurate repositioning. The same immobilization mask was subsequently used for FMISO dynamic PET scans as detailed elsewhere [10,40]. Patients were administered an intravenous bolus injection of 390 ± 16 MBq of FMISO. Approximately 300–450MBq of FDG was administered after a fasting period of ≥6 h through intravenous lines inserted in antecubital veins. The PET acquisition commenced at 70–80 min post-injection on the General Electric Discovery ST scanner (GE Health Care Inc., Chicago, IL, USA) with an imaging time of 5 min per bed position. The corresponding x-ray computed tomography (CT) images were acquired immediately prior to commencement of the PET scan and with the following settings: 140 kVp, 250 mAs, and 3.8-mm slice thickness. Each FMISO dynamic PET acquisition consisted of 3 segments: (i) at time t = 0, a 30 min dynamic acquisition binned into 6 × 5-sec, 3 × 10-sec, 4 × 60-sec, 2 × 150-sec, 2 × 300-sec, and 1 × 600-sec frames; (ii) a 10 min static acquisition, starting at ~90 min, and; (iii) a 10 min static acquisition starting at ~160 min post-injection. Between scans, patients rested in quiet waiting rooms.

### 2.3. PET/CTData Analysis

All PET data were corrected for attenuation, scatter, and random events, and were iteratively reconstructed into a 256 × 256 × 47 matrix (voxel dimensions: 1.95 × 1.95 × 3.27 mm^3^) using the ordered subset expectation maximization algorithm provided by the manufacturer. ^18^F-FDG-PET and three ^18^F-FMISO-PET scans were spatially co-registered using the rigid-body transformation calculated with the General Co-Registration TM tool applied to their corresponding CT scans (Advantage Workstation v4.7; GE Healthcare, Chicago, IL, USA). Lesions were segmented using the adaptive threshold algorithm in PET VCAR^TM^ (Advantage Workstation 4.7; GE Healthcare, Chicago, IL, USA) semi-automated software based on the companion CT as a fiduciary marker and a count-based edge recognition algorithm.

FDG uptake was calculated as the standard uptake value (SUV) corrected by lean body mass (SUL). SUV normalized by total body weight overestimates metabolic activity in all patients. Thus, the SUL is recommended for more accurate SUV results for quantitative assessment of clinical PET [20]. Tumor lesions were delineated on the FDG PET/CT images, using the adaptive threshold algorithm in the PET VCAR™ (Volume Computer-Assisted Reading; General Electric Advantage Workstation v4.7) semi-automated software, based on the companion CT as a fiduciary marker and a count-based edge recognition algorithm. The resulting segmented lesson was used to calculate the metastatic LN volumes (V_t-PET_) for PET/CT [41]. Pharmacokinetic modeling of FMISO dynamic PET data was conducted in PMOD v3.604 (PMOD Software, RRID: SCR_016547) as reported previously [10,40]. Briefly, an irreversible one-plasma two-tissue compartment model with a blood volume component was utilized to calculate surrogate biomarkers of tumor hypoxia (k_3_, tumor-to-blood ratio [TBR]), perfusion (K_1_), and total ^18^F-FMISO distribution volume (DV), i.e., the overall concentration of unbound FMISO relative to blood. Image-based input function (IF) was derived from the dynamic FMISO-PET images by segmenting the jugular vein on the early frame with the highest image intensity and fitting the time-activity curves with a triphasic exponential function.

### 2.4. MRI Data Acquisition

HNSCC patients underwent MRI examinations on a 3 Tesla (T) MRI scanner (Philips Ingenia; Philips Healthcare, Eindhoven, Netherlands) using a neurovascular phased-array coil. The standard MR multiplanar (axial, coronal, and sagittal) T_2_-weighted (T_2_w) and T_1_-weighted images were acquired as detailed elsewhere [23,42]. DW- and DCE-MRI acquisitions followed standard T_1_w and T_2_w imaging. The total MR acquisition time was approximately 30 min for the whole examination.

DW-MRI data were acquired using a single-shot echo-planar imaging (SS-EPI) sequence with the following MR parameters: repletion time (TR)/echo time (TE) = 4000/minimum (80) ms, NA = 2, matrix = 128 × 128, FOV = 20–24 cm, slices = 8–10, slice thickness = 5 mm, and ten b-values (i.e., b = 0, 20, 50, 80, 200, 300, 500, 800, 1500, and 2000 s/mm^2^). The spatial saturation bands were graphically prescribed on scout images by the technologist prior to DW-MRI scanning. Their angulation, center, and width were adjusted, depending on the neck anatomy of the patients. The total acquisition time was 5 min.

The T_1_w images for both pre-contrast (T_10_) and dynamic (i.e., before, during, and after an injection of CA) were acquired using a fast 3D T1w spoiled gradient recalled echo sequence. The pre-contrast T_1_ images were acquired with the multiple flip angles (FA) of 5°, 15°, and 30° with TR/TE = 7/2.7 ms; acquisition matrix = 256 ×1 28, FOV = 20–24 cm, slice thickness = 5 mm, and slices = 8–10. Dynamic series images were acquired using FA = 15° and other acquisition MR parameters, as mentioned above. A bolus of 0.1 mmol/kg Gd-based CA was injected through an antecubital vein catheter at two cc/s, followed by a 20-mL saline flush after acquiring 5–6 images as detailed elsewhere. A total of 40 dynamic images were obtained with a temporal resolution ranging from 7.20–8.96.0 s/image.

### 2.5. MRI Data Analysis

#### 2.5.1. DWI Analysis

DW signal intensity data from multiple b-values were fitted to (i) a monoexponential (Equation (1)) and (ii) bi-exponential equation of the IVIM model (Equation (2)) [20]:

(1) $$S\left(b\right)=S_{0}\;e^{-b\times ADC}$$

(2) $$\;\;\;\;\;\;\;\;\;\;\;\;\;\;\;\;\;\;\;\;\;\;\;\;S\left(b\right)=S_{0}\left[fe^{-b\times D^{*}}+\left(1-f\right)e^{-b\times D}\right]$$

where *S*_0_ and *S_b_* are the signal intensities without and with diffusion weighting, *b* is the diffusion weighting factor (s/mm^2^), *D* (mm^2^/s) is the true diffusion coefficient, D* (mm^2^/s) is the pseudo-diffusion coefficient (mm^2^/s), and f is the perfusion fraction. 

#### 2.5.2. Fast Exchange Regime DCE-MRI Analysis

The longitudinal relaxation rate constant-with time course for tissue *R*_1*t*_ (*R*_1*t*_ = 1/T_1t_) and EES R_1e_ in the fast exchange limit is given by Equations (3) and (4) as follows [43]:

(3) $$R_{1t}\left(t\right)=R_{10}+r_{1}\left(t\right)C_{t}\left(t\right)$$

(4) $$R_{1e}\left(t\right)=R_{10e}+r_{1}\left(t\right)C_{e}\left(t\right)$$

where *R*_10_ and *R*_10*e*_ are the precontrast longitudinal relation rate constants for tissue and EES, respectively, *C_t_(t)* and *C_e_(t)* are the CA concentration with time in tissue and EES. The r_1_ is the longitudinal relaxivity of CA.

The CA concentration with time in tissue is given by the standard Toft model (Equation (5)) [24].

(5) $$C_{t}\left(t\right)=K^{trans}\overset{t}{\underset{0}{\int }}e^{-k_{ep}\left(t-\tau \right)}C_{p}\left(\tau \right)\;d\tau$$

where *K^trans^* is the volume transfer constant, *C_p_* is the plasma CA concentration (called arterial input function [AIF]), and *k_ep_* (*k_ep_* = *K^trans^*/v_e_) is the CA transfer rate constant from the EES to vascular space. The CA concentration in EES is given by *C_e_(t)* = *C_t_(t)*)/v_e_.

The two-site water exchange (2SX) model (i.e., between the intracellular space [ICS] and EES) is derived from the three-site-two water exchange model formulated based on Bloch-McConnell’s, assuming a negligible vascular space [44,45]. The solution of Bloch-McConnell’s 2SX system yields two eigenvalues [46]. One of the eigenvalues represents the observable longitudinal relaxation rate R_1_ of the FXR model given by Equation (6) [16].

(6) $$R_{1t}\left(t\right)=\frac{1}{2}\left[\left(R_{1i}+k_{ie}+R_{1e}+k_{ei}\right)-\sqrt{{\left(R_{1i}+k_{ie}-R_{1e}-k_{ei}\right)}^{2}+4\;k_{ie}k_{ei}}\right]$$

where *R*_1*i*_ and *R*_1*e*_ are the ICS and EES longitudinal relaxation rate constants. The *k_ie_* (*k_ie_* = 1/*τ_i_*) and *k_ei_* are the water exchange rates between ICS and EES and vice versa. The *k_ie_* is related to P (A/v_i_), where *P* is the cell membrane water permeability coefficient, and A/v_i_ is the ratio of surface area to volume of a cell. For DCE data analysis, the *R*_1*i*_ value was set equal to that of R_10_.

### 2.6. MRI Tumor Regions of Interests Analysis

Regions of interest (ROIs) were manually delineated on the metastatic lymph nodes (LNs) on b = 0 (s/mm^2^) DW images and late phases of T_1_w dynamic images by a team of neuroradiologists based on T_1_w/T_2_w images using Image J [47]. The metastatic LN volumes (V_t-MRI_) were calculated from the T_2_-weighted images as detailed elsewhere [23]. The AIF was extracted from the carotid artery in each patient [48]. Equations (2) and (6) were fitted on a voxel-wise basis with a nonlinear least-square curve fitting method [49,50]. T_10_ values were estimated on a voxel-wise basis from the multiple angles as described elsewhere [51,52]. DW and DCE post-image processing, including parametric map generation, were conducted using MRI-QAMPER (MRI Quantitative Analysis of Multi-Parameter Evaluation Routines) [42,50].

### 2.7. Statistical Analysis

QI metric values derived from MMI (FDG-PET/CT, FMISO-PET/CT, and DW- and DCE-MRI) were reported as mean ± standard deviation (SD). Wilcoxon signed-rank test (WSRT) was performed to compare the tumor volume obtained from MMI techniques. A nonparametric measure of the correlation, the Spearman’s rank (ρ) analysis, was performed to examine the relationship among MMI-derived QI metrics. The correlation coefficient (ρ) of <0.3 was considered weak, 0.3–0.5 moderate, and 0.5–1.0 strong. The significance level was set at *p* < 0.05.

To determine how and to what extent the MMI-derived QI metrics were interconnected on the network, the CDA algorithm based on the “spin-glass” model was employed for the QI metrics whose Spearman’s rank test *p*-value was <0.05 [39]. The spin-glass is a unique community detection algorithm based on the statistical mechanics of spin around the networks [39]. The CDA-based “spin-glass” model approach splits MMI-derived QI metrics into distinct communities [53]. Links or edges heavily or sparsely connect the groups that can also reveal strong or weak, including positive or negative correlations [53]. All statistical analyses were performed using R-4.0.3 software [54].

## 3. Results

Ninety-two imaging datasets (FDG-PET/CT, FMSIO-PET/CT, DW-, and DCE-MRI) were successfully analyzed from 27 metastatic LNs. The median Karnofsky Performance Status (KPS) was 90 (range 80–90).

**Figure 1:**
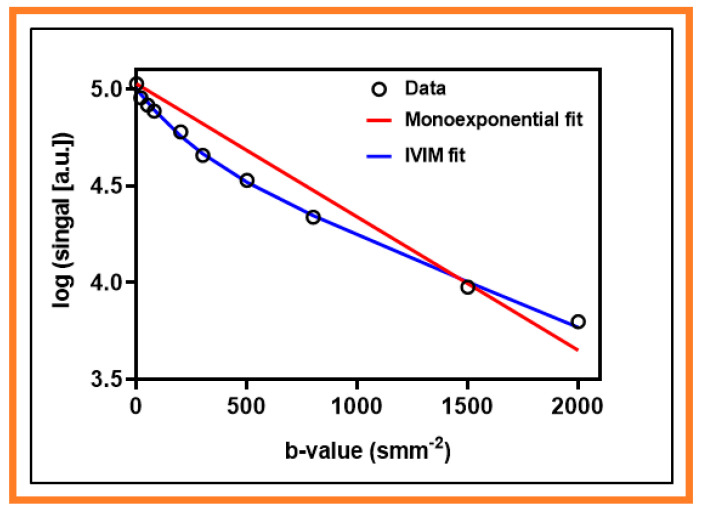
Representative mean semilogarithmic signal intensity decay curve as a function of the multiple b-value (black circle). The data were fitted with the monoexponential model (solid red line) and intravoxel incoherent motion (IVIM) model (solid blue line).

**Figure 2:**
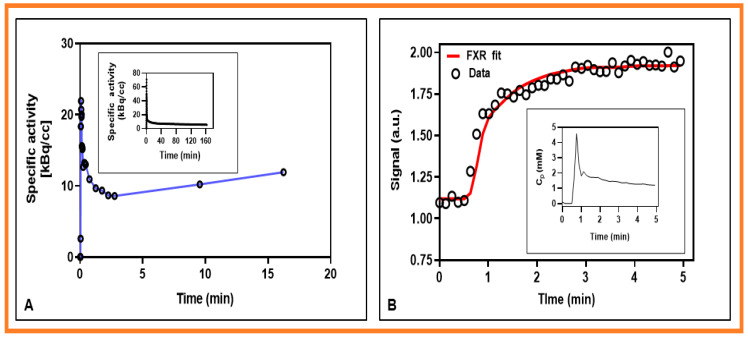
Representative multimodality imaging signal plots with time for data obtained from the metastatic neck lymph node in a patient with head and neck squamous cell carcinomas. (**A**) Measured mean FMISO-PET/CT time-activity curve (circles) data connected with a solid line (blue), and the corresponding input function (in the inset). (**B**) Signal intensity time curves fitted with the fast exchange regime (FXR) model. The circle (black) and solid line (red) represent the data and FXR model fit. Insert: Plasma contrast agent concentration, C_p_ with time.

**Figure 3:**
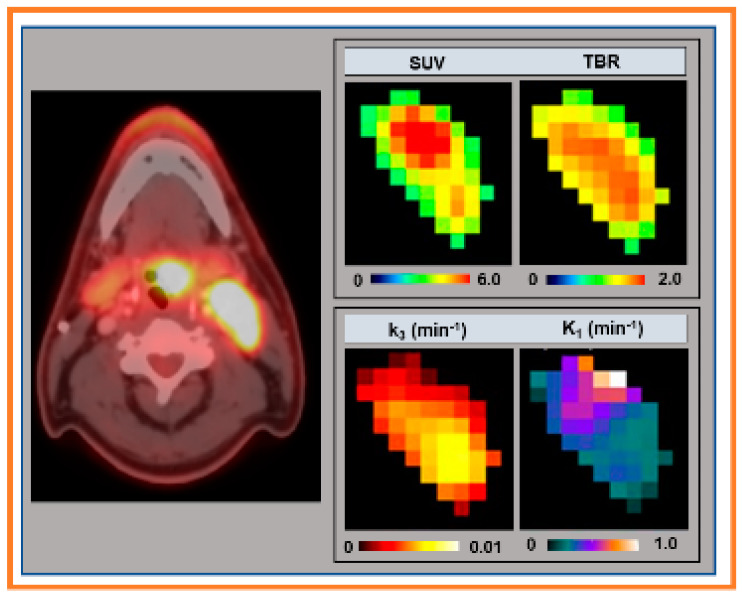
Left column: Representative axial images of ^18^F-fluoromisonidazole (^18^F-FMISO) positron emission tomography (PET/CT) from HPV-positive head and neck squamous carcinoma patient. Right column: ^18^F-fluorodeoxyglucose-PET/CT derived standard uptake value (SUV) and FMISO-PET derived tumor to blood ratio (TBR), k_3_, and K_1_ maps.

**Figure 4:**
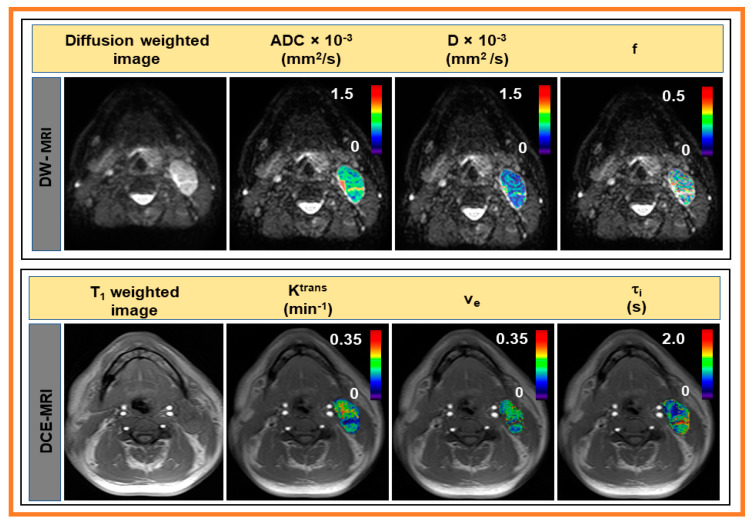
Top: Representative diffusion-weighted image (b = 0 s/mm^2^) and monoexponential and intravoxel incoherent motion models-derived parametric maps overlaid on diffusion-weighted image (b = 0 s/mm^2^). Bottom: Representative precontrast T_1_ weighted (T_1_w) image and fast exchange regime model-derived parametric maps overlaid on precontrast T_1_w images.

The representative signal versus b-values curve for DW data is displayed in Figure 1. The signal time representative curves for FMISO and DCE data are displayed in Figure 2A,B, respectively. The corresponding arterial input functions are also displayed. The FMISO data was taken from the metastatic LN displayed in Figure 3. Similarly, DW- and DCE-MRI data were extracted from ROIs shown in Figure 4. 

Figure 3 shows the representative PET/CT image and QI metrics extracted from the FDG-PET/CT and FMISO-PET/CT.

Representative DW, T_1_-weighted image, and QI metrics derived from IVIM and FXR model are displayed in Figure 4. The representative MRI images were from the same patient shown in Figure 2.

The mean tumor ROI volume and QI metrics values from MMI are given in Table 1.

**Figure 5:**
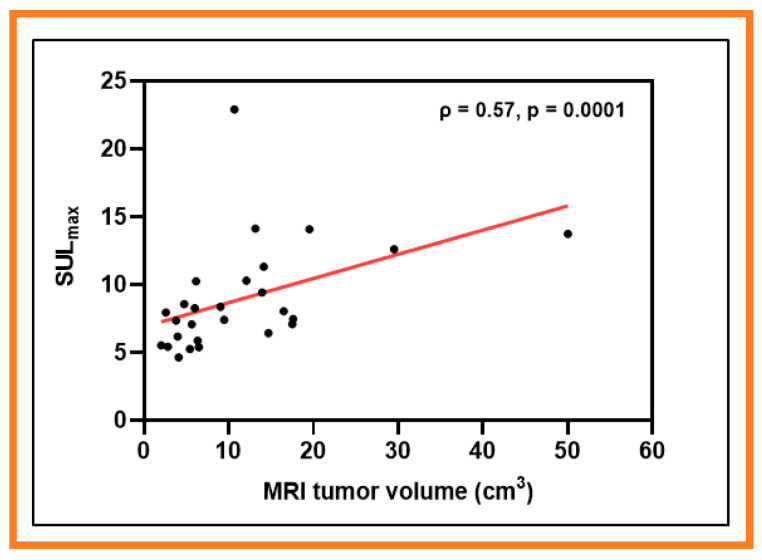
Scatter plot showing a correlation between ^18^F-FDG-PET/CT-derived maximum standardized uptake value normalized to lean body mass (SULmax) and metastatic lymph node volumes from T_2_ weighted MRI (V_t- MRI_). Circles represent the data, and the solid line denotes the line of best fit.

Mean metastatic LN volumes obtained from PET (V_t- PET_) and MRI (V_t- MRI_) were significantly different (V_t-PET_ = 13.59 ± 7.65 cm^3^ vs. V_t-MRI_ = 11.41 ± 10.09 cm^3^, *p* = 0.005, WSRT) and were strongly positively correlated (ρ = 0.85, *p* < 0.0001) in HNSCC. Mean V_t-MRI_ was strongly positively correlated with ^18^F-FDG-PET/CT-derived metrics SUL_mean_ (ρ = 0.48, *p* = 0.01) and SUL_max_ (ρ = 0.57, *p* = 0.0001) (Figure 5). The metrics SUL_max_ and SUL_mean_ derived from FDG-PET/CT represent the standardized uptake value normalized to lean body mass, respectively. No significant correlations were found between V_t-PET_ and metrics obtained from ^18^F-FMISO-PET/CT, DW-, and DCE-MRI (*p* > 0.05). V_t-MRI_ also did not show a significant correlation with ^18^F-FMISO-PET/CT, DW-, and DCE-MRI derived metrics (*p* > 0.05).

Spearman correlation analysis identified several weak, moderate, and strong statistically significant, either positive or negative, correlations between QI metrics (surrogate markers of cellularity, glucose metabolism, perfusion, and hypoxia) derived from MMI data (Table 2). Herein, a summary of the Spearman correlation results is reported.

The metrics ADC and D, markers of tumor cellularity, exhibited a significant moderate negative correlation with SUL_mean_, a feature of glycolytic activity (ρ = −0.42, *p* = 0.03 for ADC and ρ = −0.41, *p* = 0.03 for D). Additionally, there was a significant moderate negative correlation between D and K^trans^, a marker of tumor perfsuion//permeability (ρ = −0.43, *p* = 0.03), and D and τ_i_, a maker of cell metabolic activity (ρ = −0.41, *p* = 0.04). ADC and v_e_, a leakage space for CA, showed a significant positive correlation (ρ = 0.46, *p* = 0.02). D showed a moderate negative correlation with K_1_, a measure of perfusion for FMISO (ρ = −0.40, *p* = 0.04).

The metric D*, a marker of capillary perfusion in tissue, showed a moderate positive correlation with K^trans^ and K_1_ (ρ = 0.39, *p* = 0.04 for K^trans^ and ρ = 0.40, *p* = 0.04 for K_1_). The perfusion fraction, f (the volume fraction occupied by capillaries), showed a moderate positive correlation with DV, a distribution volume of FMISO (ρ = 0.40, *p* = 0.04). In contrast, it showed a moderate negative correlation with k_3max_ (ρ = −0.40, *p* = 0.02). The metrics K^trans^ and K_1_ were moderately positively correlated (ρ = 0.48, *p* = 0.01). K^trans^ and k_3max_, a marker of tumor hypoxia, were moderately negatively correlated (ρ = −0.41, *p* = 0.03). K^trans^ and DV exhibited a moderate positive correlation (ρ = 0.44, *p* = 0.02). The metric v_e_ and DV were moderately positively correlated (ρ = 0.40, *p* = 0.04).

**Figure 6:**
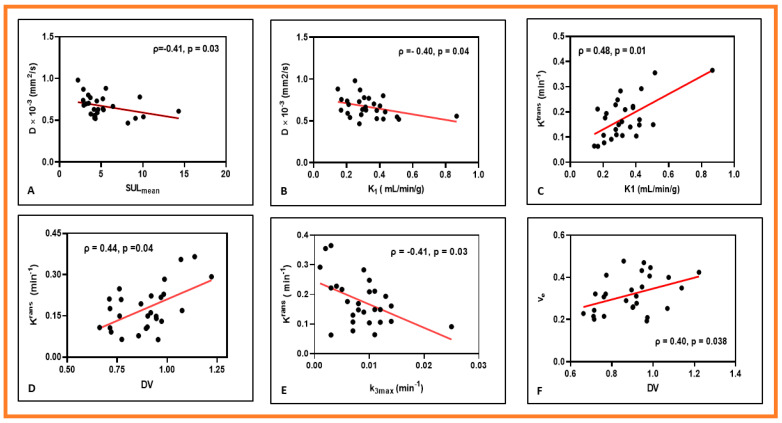
Representative scatter plots showing the relationships between quantitative imaging metrics derived from multimodality imaging methods. (**A**) True diffusion coefficient (**D**) vs. mean of the maximum standardized uptake value normalized to lean body mass (SULmean). (**B**) D vs. K_1_ (Perfusion constant for FMISO). (**C**) K^trans^ (Volume transfer constant for Gd-based contrast agent) vs. K_1_. (**D**) K^trans^ vs. DV (FMISO distribution volume). (**E**) K^trans^ vs. k_3max_. (**F**) v_e_ (Volume fraction of the extravascular extracellular space ) vs. DV.

Figure 6 shows the representative scatter plots between MMI-derived QI metrics.

**Figure 7:**
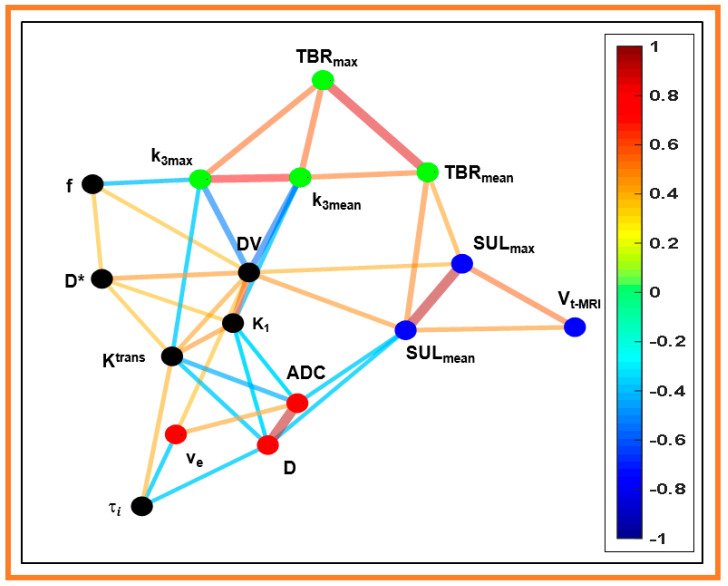
Sample network with 16 nodes and 33 edges constructed from a community detection algorithm (CDA) based on the spin-glass model. The nodes represent the quantitative imaging (QI) metrics derived from four multimodality imaging which are connected by lines or edges in the graph. The perfusion metric, K_1_, and distribution volume (DV) derived from the ^18^FMISO-PET/CT overlap with DW- and DCE-MRI metrics and are densely interconnected compared to other metrics in the network ( blue color: 3 nodes [V_t-MRI_, SULmax, mean], green color: 4 nodes [TBR_max_, mean, and k_3max,mean_] black color 6 nodes [K_1_, DV, D* and f, K^trans^ and τ_i_], and red color: 3 nodes [ADC, D, and v_e_]). A solid blue line represents the negative correlation, whereas the orange and red colors represent the positive correlation among the QIs. The thickness of lines indicates the extent of a correlation (weak, moderate, and strong). The value in the color bar scale represents either negative or positive links detected by the CDA approach in networks.

The network as a graph constructed from 16 MMI-derived QI metrics, including T_2_ weighted tumor volume (V_t-MRI_), using the CDA-based “spin-glass” analysis, is illustrated in Figure 7. The CDA approach resulted in four communities with 33 edges in the network. As a note, the edges were constructed between the nodes that yielded a Spearman rank correlation value *p* < 0.05 (Table 2). The nodes within the community are densely coupled to each other. In contrast, these nodes are relatively sparsely connected with other communities in the same graph. The solid blue line represents the negative correlation. In contrast, the positive correlation is represented by the orange and red colors, respectively. The thickness of the lines representing the extent of correlation ranging from weak to strong between them.

V_t-MR_ is a community member in the network formed by FDG-PET/CT-derived metrics (SUL_max,mean_ [3 nodes, blue color]). The quantitative metrics ADC, D, and v_e_ formed the 2nd community network (3 nodes, red color). The metrics k_3max,mean_, and TBR_max,mean_ created the 3rd community (4 nodes, green color). The fourth community consisted of 6 nodes (black color), including K_1_ and DV (FMISO), f and D* (DW-MRI), and K^trans^ and τ_i_ (DCE-MRI). CDA approach yielded 8, 7, and 6 edges for the metrics DV, K^trans^, and K_1_, respectively.

K^trans^ is regarded as one of the most influential metrics to assess tumor microvasculature. K^trans^ (a member of the black community) exhibits distinct relationships with nearby nodes (red community [ADC and D] and green community [k_3.max_]). In contrast, K^trans^ did not directly link FDG-PET/CT-derived metrics and tumor volume in the network. The extent of the K^trans^ relationship with nearby nodes (K_1_, DV, k_3.max_, and D*) was further evaluated using regression analysis. The combination of three nodes (K_1_, DV, and k_3max_) yielded a significant correlation (R^2^ = 0.45, adjusted R^2^ = 0.38, *p* = 0.002). In contrast, linear regression analysis between K^trans^ and k_3max_ yielded a meaningful inverse relationship (R^2^ = 0.23, adjusted R^2^ = 0.19, *p* = 0.011), indicating the influence of vascular permeability on radiotracer diffusion in hypoxic tumors.

## 4. Discussion

Integration of metabolic (^18^F-FDG-PET/CT and ^18^FMISO- PET/CT) and functional imaging (DW-, and DCE-MRI) aggregate the complementary information of tumor physiology [10,23,41,42]. The present study investigated a correlation at pre-TX between QIs obtained from MMI characterizing tumor = physiology, including glucose metabolism, perfusion, hypoxia, pseudo-diffusion in the capillary network, cellularity, and perfusion/permeability and metabolic activity markers using a CDA-based “spin-glass model” in addition to Spearman correlation in HNSCC patients. The CDA approach identified the four communities and revealed that K^trans^, a measure of perfusion/vascular permeability, links to seven edges in the community. Thus, detecting communities and identifying their relationship is an essential step to investigate robust imaging biomarkers for precision medicine in HNSCC. Our previous two separate studies used ^18^F-FMISO dynamic PET (dPET) to assess the tumor hypoxia and perfusion and monitor early response to chemo-RT in HNSCC [41]. The first study with ^18^F-FMISO dPET data provided parametric maps of tumor hypoxia, perfusion, and radiotracer distribution volume, improving the characterization of a tumor lesion [40]. The other study concluded that kinetic modeling of FMISO dPET data reveals a more detailed description of the tumor microenvironment and improved assessment of response to chemo-RT than a single static image [10]. In DW- MRI, IVIM derived QI metrics obtained at pre-TX and intra-TX weeks 1, 2, and 3 were used to characterize and monitor response to chemo-RT. Hierarchical clustering performed using the intra-TX IVIM derived QI metrics demonstrated the subtypes in HPV + patients in HNSCC [23].

Jansen et al. found a correlation between tumor volume and ^18^F-FMISO SUV in 13 HNSCC patients [36]. The present study also found a strong correlation between pre-TX MRI tumor volume with SUL_max,mean_. A moderate negative correlation between SUL_mean_ and ADC was consistent with previous studies in HNSCC [6,55]. FDG-PET/CT-derived SUV and DWI-MRI-derived ADC represent different aspects of the tumor cells. The glycolytic activity of tumor cells (reflected by FDG uptake) is related to high tumor cell density, consequently restricting water molecule diffusion and lowering ADC values. It has been reported that there was a strong negative correlation between the mean of pre-TX ADC and ^18^F-FDG PET SUV [56]. The present study did not find a significant positive correlation between K_1_ and _SULmax,mean_. In contrast, Vidiri et al. reported a significant negative correlation between K^trans^ and ^18^F-FDG-PET/CT parameters in LNs of oropharyngeal squamous cell carcinoma [13]. Surov et al. reported a moderate positive correlation between SUVmax and k_ep_ [11]. In contrast, in the present study, SUL exhibited a nonsignificant negative correlation with k_ep_ derived from the FXR model. Chen et al. study concluded that qMRI could provide additional value in distinguishing metastatic nodes, particularly among small nodes, when used with FDG-PET [53].

Dynamic FMISO-PET/CT and DCE-MRI-derived QI metrics capture the tumor physiology through various mechanisms. The molecular weights of FMISO (189.14 Daltons) and Gd-based CA (Gd-DTPA ~ 547 Daltons) are different, and the transport mechanism across the vasculature is assessed differently. The FMISO is lipophilic, and its uptake is driven by passive diffusion out of the vasculature and through the cell membranes. The radiotracer eventually accumulates in hypoxic regions, largely independently of the perfusion. In contrast, the K^trans^ measures the influx of a CA entering the EES, altering the T_1_ relaxation of the tissue water protons, but CA does not enter the cell. As *τ*_i_ is associated with cell size, *τ*_i_ = [(v_i_/A) × 1/*P*], where v_i_/A is the ratio of volume to the cell’s surface area and P is the cell membrane’s permeability. Thus, a decrease in metric *τ*_i_ value associated with shrinkage of a cell would correspond to an increase in ADC/D values. Previous studies reported the inconsistent correlation between τ_i_ and SUV in breast cancer [57] and hepatocellular carcinoma [58]. As a note, τ_i_ is mainly representing a cell metabolic activity characterized by adenosine triphosphate production [30]. In the present study, K^trans^ and k_3max_ exhibited a strong negative Spearman correlation.

Identifying the precise dose (e.g., dose escalation or de-escalation) for HNSCC patients is difficult because of heterogeneous populations of various disease sites, stages, and prognoses. HPV+ oropharyngeal cancer is a distinct biologic entity that shows a favorable prognosis with standard chemo-RT [59]. In contrast, HPV-negative tumors continue to have a poor prognosis despite treatment intensification. Thus, patient selection is vital for treating less aggressive radiotherapy regimens to maintain excellent standard therapy outcomes [60]. The utilities of MMI-derived biomarkers have been considerably improving tumor delineation accuracy, subvolume determination, longitudinal tracking of treatment response, permitting dose escalation or de-escalation to target tissues, and reducing toxicity to nearby tissues and organs [16], thus highlighting the need for robust biomarkers to be included in clinical trials.

Despite the significant advances in MMI methods, findings of reliable biomarkers that can effectively assess changes in tumor physiology after RT are still challenging, especially for precision therapy in HNSCC, given that we do not yet know which one of these imaging modalities is the gold standard. The present study CDA “spin-glass”-based analysis resulted in four communities for 16 MMI-derived metrics, clustering related metrics together in a network. This indicates a preferential linking between nodes to the other groups in the network exhibiting similar characteristics [39]. K_1_ and K^trans^, measures of the tumor perfusion and permeability for FMISO and Gd-based CA, showed a strong connection in the CDA network. Similarly, DV and v_e_, distribution volume for FMISO and CA, exhibited a similar relationship. In contrast, k_3max_, a hypoxia marker, was negatively correlated with the K^trans^ and f. The metric k_3_ is related to the diffusive compartment, which is hypoxic, consistent with the view that tumor hypoxia results from inadequate oxygen supply to the tumor [61]. As a note, K^trans^ represents a passive transport of CA across the capillary wall driven by diffusion, whereas k_3max_ is the FMISO uptake in hypoxic tissues caused by convective transport [62]. Therefore, ^18^F-MISO-PET/CT and DCE-MRI can provide complementary information for characterizing the tumor microenvironments [63]. The community structure displayed by a CDA approach is visually interpretable to identify important biomarker metrics and infer their relationships. Thus, the CDA approach may improve in identifying the surrogate biomarkers for prognostication at pre-TX in HNSCC patients.

The present study is limited by the sample size for CDA analysis, which warrants validation in a larger sample. Motion artifacts in the MR images due to the voluntary and involuntary movements in the neck region, such as swallowing and breathing, can be minimized by carefully setting up the scan. Respiratory motion artifacts can be minimized with proper breath-holding and shortened scan duration. A robust co-registration method could improve the correlation between QIs. The present study was also limited to assessing the correlation between mean QI values rather than a voxel-by-voxel basis. A B_1_ non-uniformity due to varying the flip angle influences the accuracy and precision of DCE-MRI-derived QIs at higher field strength. Hence, acquiring the B_1_ mapping sequence can improve the accuracy of DCE-MRI-derived QI metrics. Despite these limitations, the CDA approach demonstrated its potential in assessing the correlation between the QI metrics.

## 5. Conclusions

Significant Spearman correlations, ranging from moderate to strong, were observed between few QI metrics. The CDA approach illustrated how and to what extent MMI-derived QI metrics were associated in the network. After validation in a larger HNSCC population, the present preliminary findings may help identify potential biomarkers in individualized patient care.

## Figures and Tables

**Table 1:** Summary of multimodality imaging derived quantitative imaging metrics values.

Method	Model Parameter	Value ^1^
^18^F-FDG-PET/CT	SUL_max_	8.91 ± 3.94
	SUL_mean_	5.26 ± 2.76
^18^F-FMISO-PET/CT	K_1_ (min^−1^)	0.33 ± 0.15
	k_3 max_ (min^−1^)	0.0087 ± 0.0049
	k_3 mean_ (min^−1^)	0.0034 ± 0.0021
	TBR_max_	1.76 ± 0.53
	TBR_mean_	1.29 ± 0.27
	DV	0.89 ± 0.14
DW	ADC × 10^−3^ (mm^2^/s)	0.93 ± 0.14
	D × 10^−3^ (mm^2^/s)	0.67 ± 0.13
	D* × 1 0^−3^ (mm^2^/s)	9.02 ± 1.80
	f	0.16 ± 0.06
DCE	K^trans^ (min^−1^)	0.18 ± 0.06
	v_e_	0.32 ± 0.09
	τ_i_ (s)	0.670 ± 0.15
FDG-PET tumor volume	V_t-PET_ (cm^3^)	13.59 ± 7.65
T_2w_ MRI tumor volume	V_t-MRI_ (cm^3^)	11.41 ± 10.09

Note: ^1^ Data are represented as mean ± SD.

**Table 2:** Summary of Spearman rank correlation analysis (ρ) results between the quantitative metrics derived from multimodality imaging data.

Quantitative metric	PET volume	MR T_2_ weighted volume	ADC	D	D*	f	K^trans^	v_e_	τ_i_	k_ep_	SUL_max_	SUL_mean_	K_1_	k_3.max_	k_3.mean_	DV	TBR Max	TBR Mean
**PET volume**		0.84 *	−0.09	−0.10	0.28	−0.06	0.19	−0.09	0.16	0.17	0.58 *	0.42 *	0.26	0.29	0.13	0.16	0.30	0.20
**MRI T_2_ weighted volume**			−0.17	−0.18	0.07	0.01	0.14	−0.14	0.12	0.26	0.57 *	0.48 *	0.17	0.30	0.31	0.04	0.25	0.27
**ADC**				0.95 *	−0.34	−0.18	−0.48 *	0.46 *	−0.36	0.18	−0.29	−0.42 *	−0.40 *	0.26	0.15	−0.31	0.07	−0.14
**D**					−0.26	−0.18	−0.43 *	0.32	−0.41 *	0.23	−0.31	−0.41 *	−0.40 *	0.32	0.23	−0.32	0.14	−0.08
D*						0.39 *	0.39 *	−0.11	0.01	0.04	0.21	0.24	0.40 *	−0.25	−0.35	0.49 *	0.09	0.13
**f**							0.31	0.18	0.14	−0.14	0.06	0.20	0.20	−0.45 *	−0.30	0.40 *	−0.18	0.12
**K^trans^**								−0.10	0.43 *	0.12	0.17	0.28	0.48 *	−0.41 *	−0.27	0.44 *	−0.17	−0.03
**v_e_**									−0.41 *	−0.15	0.17	0.15	0.02	−0.20	−0.26	0.40 *	0.14	0.13
**τ_i_**										−0.03	0.06	0.11	0.08	−0.17	0.05	−0.04	−0.23	−0.12
**k_ep_**											−0.11	−0.16	0.20	0.34	0.20	−0.23	−0.09	−0.20
**SUL_max_**												0.94 *	−0.01	−0.09	−0.02	0.42 *	0.36	0.44 *
**SUL_mean_**													0.003	−0.15	−0.02	0.48 *	0.38	0.53^*^
**K_1_**														−0.26	−0.46 *	0.59 *	−0.18	−0.19
**k_3.max_**															0.79 *	−0.57 *	0.57 *	0.34
**k_3.mean_**																−0.60 *	0.57 *	0.54 *
**DV**																	0.13	0.23
**TBR_max_**																		0.88 *
**TBR_mean_**																		

The *p*-value < 0.05 is denoted by an asterisk *. ADC: Apparent diffusion coefficient, D: true diffusion coefficient, D*: pseudo-diffusion constant, f: perfusion fraction, K^trans^: volume transfer constant, v_e_: volume fraction of extravascular extracellular space (EES), τ_i_: mean lifetime of water molecules, k_ep_: transport constant for contrast agent form EES to blood plasma space, SUL: standardized uptake values of ^18^F-fluorodeoxyglucose divided by lean body mass, K_1_: transport rate constant of tracer from the plasma to the tissue for ^18^F-fluoromisonidazole (FMISO), k_3_: kinetic rate constant approximating the rate of irreversible binding of FMISO, TBR: Tumor-to-Blood Ratio, and DV: total ^18^F-FMISO distribution volume.

## Data Availability

The data presented in this study will be provided upon reasonable request.
